# Retention Capacity of Original Denture Adhesives and White Brands for Conventional Complete Dentures: An In Vitro Study

**DOI:** 10.3390/polym14091749

**Published:** 2022-04-26

**Authors:** Joana Mendes, José Manuel Mendes, Pedro Barreiros, Carlos Aroso, António Sérgio Silva

**Affiliations:** UNIPRO—Oral Pathology and Rehabilitation Research Unit, University Institute of Health Sciences (IUCS), CESPU, 4585-116 Gandra, Portugal; a27209@alunos.cespu.pt (J.M.); pedro.barreiros@iucs.cespu.pt (P.B.); carlos.ribeiro@iucs.cespu.pt (C.A.); asergio.silva@iucs.cespu.pt (A.S.S.)

**Keywords:** denture adhesives, adhesion mechanism, saliva, complete denture, denture retention, oral health-related quality of life, alveolar ridge

## Abstract

(1) Introduction: Denture adhesives (DAs) promote stability, chewing ability, and quality of life. The objective of this study was to compare the effectiveness of original brand DAs and white brands in their retention of conventional complete dentures. (2) Methods: This study followed the recommendations of the international standard ISO 10873. Three original brands of DA (Corega^®^ Fixação 3D, GlaxoSmithKline^®^, Stafford Miller Ltd., Dungarvan Co. Waterford, Ireland), KuKident^®^ Pro Procter & Gamble Technical Centres^®^, Ltd., Whitehal Lane, Germany and Elgydium^®^ Fix, Laboratórios URGO^®^ SL, Florida, Spain) were compared to three white brands (Fixação Extra Forte Pingo Doce^®^, Laboratórios Cosmodent^®^, Cantabria, Spain), Fixador de Próteses (Continente^®^, Propack^®^,Gmbh, Ladenburg, Germany) and Creme Fixador de Próteses (Auchan^®^, Ellipse^®^, Roubaix, France). Their retention capacities were analyzed using a mechanical test device. (3) Results: The mean retentive ability of original brand adhesives (M = 11.16, SD = 5.27) was significantly higher (t(298) = 11.88; *p* < 0.001) than that of the white brands (M = 5.92, SD = 1.18). When comparing all brands, statistically significant differences were also observed, F(5.294) = 707.68 (*p* < 0.001). The generic adhesive results were more homogeneous. The generic brands from Continente^®^ (M = 5.24, SD = 0.94) and Auchan^®^ (M = 5.80, SD = 0.79) were not significantly different, while the Pingo Doce^®^ brand obtained significantly higher mean retention results (M = 6.71, SD = 1.28). (4) Conclusions: The original brands of DA have a significantly higher retentive ability than the white brands. Elygidim^®^ Fix had the worst result of the three original brands, and the product from Pingo Doce^®^ had the best result among the three white brands.

## 1. Introduction

With the aging of the population, the number of elderly people in developed countries is predicted to reach 400 million by 2050 [1]. Oral health is particularly important in the elderly, and medical professionals should be prepared to address their dental problems [2]. Many countries have reorganized their dental services to meet this need [3], as it can improve the quality of life of their geriatric population [4,5,6].

Total prostheses are used to treat edentulous patients. The stability and retention of these prostheses can be achieved with dental implants or by adhesion to the oral mucosa. Conventional complete dentures (CDs) remain the first choice, especially in older patients, due to economic factors [7]. 

Rehabilitation with total maxillary protheses is usually well tolerated by patients, as compared to total mandibular prostheses. The main source of dissatisfaction with total mandibular prostheses has been identified as lack of retention and stability [8,9].

Retention and stability are influenced by mandibular atrophy and by the movement of the tongue during mastication and phonation. The problems of retention are often correlated with loss of bone support, due to reabsorption of the alveolar ridge of the mandible, requiring a more complicated prosthetic treatment, as compared to that of the maxilla [10].

Clinical and laboratory studies concerning the retention of total prostheses have examined the placement of endosseous implants to ensure retention for patients with total prostheses [11,12,13]. Other studies have examined non-surgical methods, such as the use of denture adhesives (DAs) [8]. 

Adhesive placed on prostheses is one of the methods used to avoid the disinsertion of the removable prostheses of the edentulous. This promotes primary retention of the prostheses, which may be difficult, or impossible, to obtain via other methods. DAs are sold in the form of gels, powders, creams, and adhesive strips, and are placed between the base of the prostheses and the oral mucosa [8,14]. DAs improve the patient’s quality of life as a result of their increased satisfaction and the resultant positive psychological impact. They promote stability, mastication, and better adhesion until the removal of the prostheses [14,15,16]. They are composed of short- or long-chain synthetic polymers.

The usual components are carboxymethyl cellulose (CMC) and polyvinyl methyl ether/maleic acid (PVM-MA), antimicrobial agents, and plasticizing or flavoring agents [17,18].

The retention of removable total prostheses in the oral cavity is accomplished by adaptation of the supportive tissues between the mucosa and the base of the prostheses, through the thin layer of saliva between them, and with atmospheric pressure. Saliva, which hydrates these materials, increases the volume by occupying the space between the base of the prostheses and the oral mucosa and improving retention. Better adhesion of the prostheses to the oral mucosa is more comfortable for patients, as it reduces the medio-lateral movements of the prostheses and promotes stronger bite force [17,18,19,20]. The retention of the CD is important, as a lack of retention may induce movement in the base of the prosthesis due to deformation of the oral mucosa [11,21,22].

Due to the reduced alveolar ridge in edentulous patients, which is associated with the volume of saliva necessary for the retention of the removable total prostheses, DAs assure stability during masticatory and phonetic cycles and, thereby, improve the patient’s quality of life [23,24,25].

Studies have been conducted to assess the retention of total prostheses using endosseous implants and coating materials, as well as the application of dental adhesives for the retention of total prostheses. Clinical investigations have been conducted in vivo and in vitro. The retention of mandibular prostheses is influenced by reduced and mobile crests and lingual interference, as well as the entire oral musculature [10,13,26].

For an in vitro study to be objective, we must consider the mechanical factors that interfere with prosthesis retention. Laboratory tests should be conducted to simulate the real situation. In vitro tests increase our understanding of mechanical factors that affect the retention of mandibular dentures. In the present study, we included the function of saliva, the strength and the speed of the loads, and a model that simulated the anatomy of the alveolar ridge with the physiological components of the oral mucosa as well as of the surrounding tissues. An in vitro study should be designed to reproduce the environment of the oral cavity. This type of test, considering its similarity to the real environment, also ensures more reliable clinical trials. In vitro tests can control mechanical factors that can lead to lack of retention of total removable prostheses [20,26,27,28].

We developed a model that reproduced the physical properties of mandibular oral tissues to test the influence of dental adhesives, according to several previous studies [26,28]. The technique and materials used were described by Johnson et al., [27] but the modifications performed in this study were based on new technologies and laboratory evidence, as well as other studies [29,30]. 

The main objective of this study was to compare original and white DAs, using three brands in each category, in a mandibular in vitro simulation model, which reproduced the environment and the physical properties of oral tissues. Secondly, it was our goal to determine which DA had the highest retention power among the original brands and white adhesives, to assess whether there were differences in retention between DAs with the designation “strong fixation” and “extra-strong fixation”, to determine which DAs had the most retention power in each category, and to compare the cost–benefit ratio between the various DAs.

## 2. Materials and Methods

### 2.1. Materials

In this study, all materials used were selected based on their importance and usefulness in dental medicine, as well as their stability under normal conditions of use and storage. All materials and chemicals were used and received without additional purification, unless otherwise mentioned by the manufacturers. This study followed the guidelines of the international standard ISO 10873, Dentistry-Denture adhesives [31].

#### Materials Used in the Study

The silicone coating materials used in the study model were ProGel Neutral skin ™ (Principality^®^ FX, London, UK) and Elite soft relining™ (Zhermack^®^ SpA, Badia Polesine Rovigo, Italy).

The acrylic resins used were selected due to their relevance and usefulness in dental medicine and their stability under normal conditions of use and storage. The self- polymerizable acrylic resin sample consisted of a polymer, methylmethacrylate, Megacryl^®^ N (Megadental^®^ GmbH, Büdingen, Germany), and a monomer, Megacryl^®^ S + N (Megadental^®^ GmbH, Büdingen, Germany). This is a cold self-setting polymer with methyl-methacrylate-based microspheres, without tertiary amine or cadmium. It has contraction reduction and significant reduction in the residual monomer (20–30%). It has excellent fluidity, mechanical properties, natural coloring, and safe behavior, so it is stable and modulable.

The artificial saliva used in the tests was an aqueous solution based on Fusayama Meyer’s^®^ formula [32,33]. An aqueous solution containing 1000 g urea; 0.906 g CaCl_2_, 2H_2_O; 0.690 g NaH_2_PO_4_, 2H_2_O; 0.400 g KCl; 0.400 g NaCl; 0.005 g Na_2_S, 9H_2_O) was elaborated. The solution was diluted with deionized water to 1000 mL.

The dental adhesives (Das) used in the study were three original brands: Corega^®^ Fixation 3D, KuKident^®^ Pro and Elgydium^®^ Fix, and three white brands sold in supermarkets: Extra-strong Fixation (Pingo Doce^®^), Prosthesis Fixer (Continente^®^), and Prosthesis Fixer Cream (Auchan^®^), as described in Table 1. All selected DAs were classified as Type 1, Class 2, according to ISO 10873:2021 [31].

### 2.2. Methods

To test all selected samples, a standard laboratory protocol was established and applied at the Laboratory of Investigation in Oral Rehabilitation and Prosthodontics, UNIPRO Oral Pathology and Rehabilitation Research Unit, University Institute of Health Sciences (IUCS), CESPU, Gandra, Portugal.

#### 2.2.1. In Vitro Model Design

Sixty models were developed to reproduce the physical properties of mandibular oral tissues. These models were designed to characterize the strength of adhesion for dental prostheses against traction load, simulating the environment of the oral cavity. 

**Figure 1:**
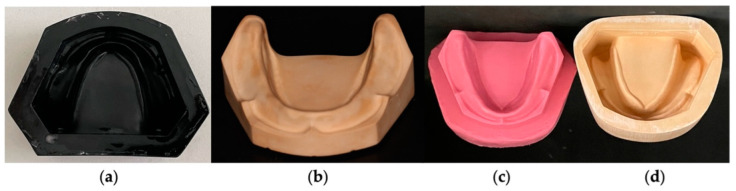
Elaboration of the work model. (**a**) Plaster model mold (Edentulous Jaw); (**b**) Duplication of the plaster model mold; (**c**) Positive mold of the silicone work; (**d**) Negative plaster mold of the original model.

The process began with the production of a work model with a reabsorbed mandibular crest and surrounding soft tissues through a Plaster Model Mold (Edentulous Jaw), Nissin^®^ (Dental Products INC, Minami-ku, Kyoto, Japan), Figure 1a. This mold was duplicated with type IV plaster Gil Rock’s^®^ (SRL Dental Gmbh, Ludwigshafen, Germany) and distilled water calibrated with a doseator (Klasse for Dental^®^ Augsburg, Germany) using a vacuum mixer Obodent Vacudent (Obodent^®^ Gmbh, Hunteburger, Germany), Figure 1b.

We made a positive mold of silicone, working from the model obtained using a formwork and duplicating it with Dublisil^®^ 20 silicone duplicator (Dreve^®^, Dentamid Gmbh, Unna, Germany), Figure 1c. The duplicated silicone mold was leaked to type IV plaster, as described above, and a negative plaster mold of the initial model was obtained, Figure 1d.

**Figure 2:**
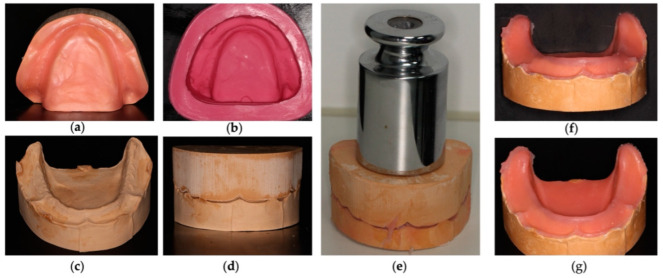
In vitro model simulating a total inferior edentulous. (**a**) Negative plaster model with wax application; (**b**) Duplication of the model with Dublisil^®^ 20; (**c**) Working model to perform mucosa simulation; (**d**) Positioning of positive and negative plaster models; (**e**) Models with characterization material awaiting adhesion with a weight of 1 Kg; (**f**,**g**) Final model of mucosa simulation.

A wax layer was applied to the negative plaster model to reduce 1 mm thickness throughout the area corresponding to the oral cavity and 3 mm of wax was applied in the crest area, to obtain a model that simulated the mucosa of a total edentulous patient with resorption of the alveolar crest, Figure 2a. Small holes were made in the wax in order to obtain space limiters between the models and to guarantee an equitable material height among all models in the study. After the wax model, a formwork was performed, and it was duplicated with Dublisil^®^ 20, Figure 2b. After the duplication model was leaked with type IV plaster, Gil Rock’s^®^, the working model was obtained to perform the simulation of the mucosa, with a reduction of 1 mm in the area corresponding to the tongue and remaining oral mucosa and with 3 mm reduction in the alveolar crest area, Figure 2c. Sixty models with reduction were obtained, according to the described methodology. The positive and negative plaster models, with the space limiters introduced, ensured a uniform thickness of mucosa simulation material in all models, Figure 2d. In the model of work with the reduction to simulate the oral mucosa and soft tissues, the filling was made with the characterization materials and silicone, namely ProGel Neutral skin ™ (Principality^®^ FX, London, UK) for the intermediate layer and Elite soft relining™ (Zhermack^®^ SpA, Badia Polesine Rovigo, Italy) for the base and surface layer. [26,27]. 

The two models were closed, and the space limiters were inserted in order to pack the simulation materials with the calculated thicknesses. With a weight of 1 Kg placed on the models for 2 h, the simulation material adhered without dimensional changes, Figure 2e. The result was an in vitro model with similar oral tissues and an alveolar crest with resilience, simulating a partial edentulous patient, Figure 2f,g. Sixty simulation models were produced according to the described methodology. 

#### 2.2.2. Preparation of the Base of Prosthetics

**Figure 3:**
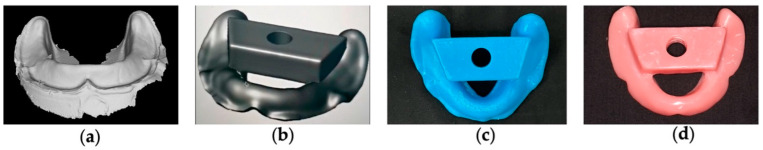
Elaboration of the prosthetic bases. (**a**) Digitalization of the final model; (**b**) Planning of the bases with Meshmixer^®^ software; (**c**) Prosthetic base printed on a Planmeca Creo^®^ C5 3D printer; (**d**) Prosthetic base in acrylic resin.

We produced ten bases of total prosthetics from the in vitro models as previously described by using a digital technique. The digitalization was executed as per the manufacturer’s instructions to ensure the correct printing of the entire plateable area of the simulation model. The printing was made with a Planmeca Emerald™ intraoral Scanner (Planmeca^®^ Oy, Helsinki, Finland), reducing the distortion, expansion, and contraction errors of the conventional impressions. The obtained digitalization was stored in Planmeca Romexis^®^ software (Planmeca^®^ Oy, Helsinki, Finland), converted into a standard tessellation language (STS) file, Figure 3a, and saved, so a 3D printed base could be developed and produced. A prosthetic base adapted to 60 simulation models and incorporating test machine support, Figure 3b, was tri-dimensionally planned with Meshmixer^®^ software. This base was elaborated according to the requisites of a total prosthesis, with insertion relief and no spacer, so as not to create resistance in the insertion and disinsertion of the base on the simulation model. The bases were printed on a Planmeca Creo^®^ C5 3D printer, Figure 3c.

The simulation base was duplicated using a muffle furnace with condensation silicon, Zetalabor^®^ (Zhermack^®^ SpA, Badia Polesine Rovigo, Italy), in order to reproduce 10 bases in acrylic resin. The bases that reproduced the conventional complete dentures (CDs) were elaborated with the self-polymerizing acrylic resin, Megacryl^®^. This acrylic resin was produced according to the supplier’s instructions. The simulation base was removed from the muffle furnace, and the acrylic resin was introduced. The muffle was placed in a pan of hot water, according to the manufacturer’s recommendations for time and temperature. We proceeded to finish and polish the CDs, Figure 3d. This process was repeated 10 times to obtain the necessary bases for the study.

#### 2.2.3. Traction Test to Measure Retention Strength of Adhesives for Dental Prosthetics

**Figure 4:**
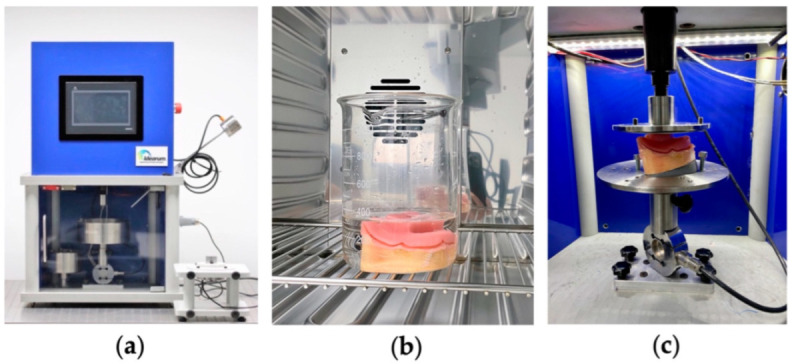
Traction Test. (**a**) Mechanical test machine CS^®^ Dental Testing Machine (Barcelona, Spain); (**b**) Simulation model with prosthetic base after placement of dental adhesive, with pressure; (**c**) Positioning of the simulation model in the test machine.

An in vitro simulation model was used to compare the retention of CDs with 6 types of DAs. The CD bases were provided with a coupling that allowed them to connect to the testing machine. The adhesion strength was determined using a mechanical test machine, CS^®^ Dental Testing Machine (Barcelona, Spain), as shown in Figure 4a. The CS^®^ Dental Testing Machine is a fatigue testing device, built in accordance with 2006/42/EC machine safety and EN 12100–1/2, EM 954–1, EN 1037, EN 61310–1/2, EN 60204–1, EN ISO 14121–1, and EN ISO 13850 standards. The CS^®^ Dental Testing Machine was attached to a support table to affix the simulation model, allowing different angulations, so that all models could be adjusted and produce equal traction. This way, the bases and the models were parallel to the transportation table. The test condition was based on the ISO 10873 norm [31], for dental prosthetic adhesives type 1, class 2. For the study, an aqueous solution of artificial saliva was prepared, based on the Fusayama Meyer formula. Artificial saliva was used because the use of human saliva for in vitro studies is limited by its lack of stability outside of the oral cavity and by colonization with bacteria [32]. For each adhesive, there were 10 simulation models and 10 CD bases, so that each adhesive was tested 10 times. A total of 10 test units for the 3D Fixation Corega^®^ adhesive; 10 test units for the KuKident^®^ Pro adhesive; 10 test units for the Elgydium^®^ Fix adhesive; 10 test units for the Fixação Extra Forte (Pingo Doce^®^) adhesive; 10 test units for the Fixador de Próteses (Continente^®^) adhesive; and 10 test units for the Crème Fixador de Próteses (Auchan^®^) adhesive.

The dental adhesive retention tests were performed according to the International Standards Organization (ISO) 10873:2021 (Dentistry, Denture adhesives) norm. The tests were all conducted at a temperature of 23 ± 3 °C, which was maintained during the entire procedure.

The adhesion strength test II, for type 1, class 2 denture adhesives was made according to the ISO norm. A total of 200 mg of adhesive was applied to the inside of the CD bases, which were placed on the simulation models. The simulation model/prosthetic base ensemble was dipped in 300 mL of saliva for 10 min and kept in an incubator (Memmert^®^, Germany) at 37 °C to simulate the environment of the oral cavity, Figure 4b. The combined simulation model/prosthetic base was removed from the incubator and shaken to remove water from the surface, and then it was installed on the support base of the mechanical testing machine, so that there was no interference in insertion and disinsertion. A load of 10 N was applied at a cross-head speed of 5 mm/min, with the axis sensible to pressure (piston), so that the load was applied on the whole of the sample. The load was kept in position for 30 s to simulate the occlusal load, Figure 4c. The base was then pulled in the opposite direction at a cross-head speed of 5 mm/min. The maximum measured force sensitive to pressure was registered, and the retention strength was calculated. The traction test was repeated four more times to obtain five results.

The number of samples used for each test group was 10 replicas (10 bases and 10 simulation models) for each adhesive. This test was repeated 10 times, and the retention strength of each DA was measured 50 times, according to the models made for each DA, for a total of 150 repetitions for each type/brand (white label vs. original). The results of the tests were copied to a Microsoft Office Excel spread sheet, for performance of statistical analysis of the data.

### 2.3. Statistical Analysis

Data were analyzed with R, version 4.1.2 [33]. Continuous distributions were assessed with histograms, symmetry and kurtosis coefficients, and Kolmogorov–Smirnov normality tests. Homogeneity of variances was evaluated with Levene’s test. Considering the results of the tests and the sample size, parametric tests were implemented. Retentive abilities of the adhesives were described as means (M) and standard deviations (SD), complemented with boxplots. Points corresponding to the sampling observations were added to boxplots. To avoid overlap of observations in the chart, a jitter function was added to the distribution. The retentive ability of the adhesives was compared by the type of brand and type of fixation, using *t*-tests and analysis of variance (ANOVA). The ANOVAs were complemented with Tukey’s tests for multiple comparisons. The interaction between fixation type and brand type was evaluated with a factorial ANOVA. The association of price increment with adhesive retention was evaluated with a generalized linear model, having as a baseline the mean of retention obtained in white label brands, considering an approximate price of EUR 3.30. The prices of the original brands were included by considering the midpoint of their price range. To evaluate the cost–benefit of the price in relation to the mean gain of retention, non-standard coefficients, and their 95% confidence intervals, were calculated. Model quality was assessed with R2. The level of significance considered for rejection of the null hypothesis was 5%.

## 3. Results

**Figure 5:**
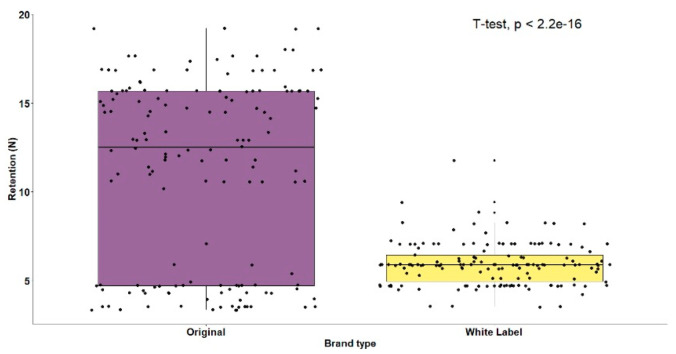
Boxplots for adhesive retention by type of brand.

**Figure 6:**
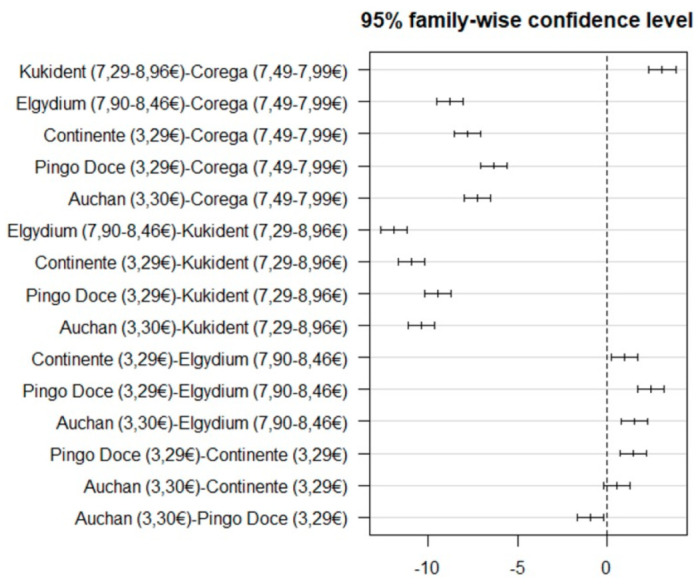
Tukey multiple comparison tests for adhesive retention capacity between all brands.

The mean retentive ability of original brand adhesives (M = 11.16, SD = 5.27) was significantly higher (t(298) = 11.88; *p* < 0.001) than white label ones (M = 5.92, SD = 1.18) (Table 2, Figure 5). When all brands were compared, statistically significant differences were also observed, F(5.294) = 707.68 (*p* < 0.001), as shown in Table 2. The white label adhesive results were more homogeneous. The brands Continente^®^ (M = 5.24, SD = 0.94) and Auchan^®^ (M = 5.80, SD = 0.79) were not significantly different, while the Pingo Doce^®^ brand obtained significantly higher mean retention results (M = 6.71, SD = 1.28) than the other two brands (Figure 6). The original brand adhesives showed higher retention differences, namely the Elgydium^®^ Fix brand (M = 4.27, SD = 0.76), which obtained the lowest mean retention values of the entire study, with significant differences to all other brands, original and white label (Figure 6). The KuKident^®^ Pro brand (M = 16.16, SD = 1.34) had the highest mean retention capacity of the study, with statistically significant differences, as compared to all other brands, including the Corega^®^ Fixação 3D brand (M = 13.05, SD = 2.13), which obtained the second highest result that was also statistically significant, as compared to all other brands (Figure 6).

**Figure 7:**
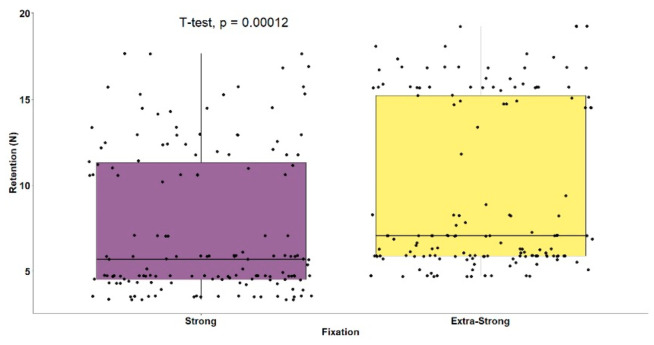
Boxplots for comparing adhesive retention by type of fixation.

Table 3 and Figure 7 show the comparisons of adhesive retentive ability by fixation type and brand. The group of extra-strong fixation adhesives had mean retention results (M = 9.57, SD = 4.84) significantly higher, t(298) = 3.90 (*p* < 0.001), than the group of strong fixation adhesives (M = 7.52, SD = 4.19). The brands KuKident^®^ Pro and Corega^®^ Fixação 3D obtained the highest results among the extra-strong and strong fixation adhesives, respectively, with statistically significant differences as compared to all other brands (Figure 7).

**Figure 8:**
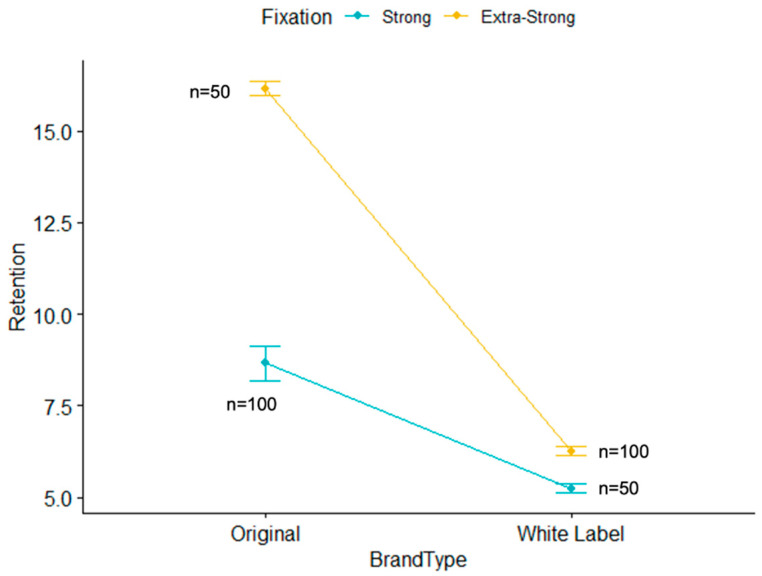
Interaction of the type of fixation with the type of brand.

Table 4 shows the results of the factorial ANOVA, including univariate comparisons of the type of fixation and brand as well as the interaction between both. This interaction was statistically significant, F_(1.296)_ = 84.83 (*p* < 0.001). As shown in Figure 8, the retentive ability of strong fixation adhesives showed smaller differences between original (M = 8.66, SD = 4.69) and white label brands (M = 5.24, SD = 0.94), as compared to extra-strong fixation adhesives, where the differences between the original (M = 16.16, SD = 1.34) and white label brands (M = 6.26, SD = 1.15) were higher.

**Figure 9:**
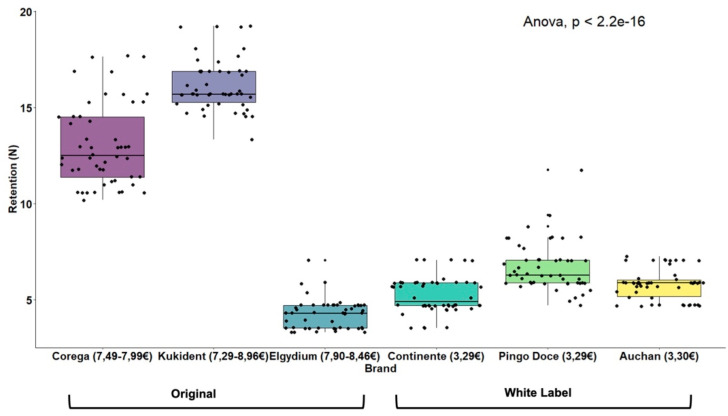
Boxplots for comparing adhesive retention by brand.

Table 5 and Figure 9 shows the cost–benefit relation of purchasing original brands, as compared to white label brands. Since the retentive ability among white label brands was not significantly different per type, the baseline category was represented by the three white label brands, with a total of 150 observations. The price-range midpoints of the original brands were calculated to measure the association of price increase with retentive ability. At baseline, with an approximate cost of EUR 3.30, it was possible to purchase a product with a mean retentive ability of 5.92 N (95%CI = [5.70; 6.14], *p* < 0.001). Paying an additional EUR 4.44 and purchasing the Corega^®^ Fixação 3D brand was associated with a mean increase of 7.14 N in retention capacity (β = 7.14, IC95% = [6.70; 7.57], *p* < 0.001), as compared to white label brands. Paying an additional EUR 4.82 and purchasing KuKident^®^ Pro brand was associated with a mean increase of 10.24 N (β = 10.24, IC95% = [9.80; 10.67], *p* < 0.001), as compared to white label brands. In contrast, paying an additional EUR 4.88 and purchasing the Elgydium^®^ Fix brand did not improve the retention capacity; instead, it revealed a decrease of 1.65 N (β = −1.65, IC95% = [−2.09; −1.21], *p* < 0.001) in the retention capacity, as compared to baseline white label brands in this model. The total variance was explained by the price/brand ratio at 91.4%.

Resume of the retention capacity of the adhesives in descending order, their characteristics, and their relation with price (Table 6):

## 4. Discussion

The increase in life expectancy at birth has led to an increase in the number of edentulous patients in the population, which has amplified the need for research concerning complete dentures.

Several studies have cited food entrapment or accumulation under prostheses as one of the most common complaints of prosthesis wearers, resulting in pain and discomfort. All of these quantitative studies reported a statistically significant reduction in food particle entrapment under the denture when an adhesive was used. These studies also reported greater comfort, confidence, and satisfaction with the prosthesis when a denture adhesive was used than when no adhesive was used. However, the ability of this methodology to discriminate between the effectiveness of different adhesive formulations or different adhesive application techniques has yet to be demonstrated. One of the challenges faced by edentulous patients is the retention of these prostheses, especially in patients with retracted or reabsorbed alveolar ridges. This is often associated with alveolar ridges with characteristics that prevent the retention of a removable prosthesis. In vitro studies can reproduce these characteristics so that the results can be extrapolated in vivo [30,31,32,33,34].

Kore et al. found that some of the missing aspects in their study were keratinized mucosa and saliva, which influence the support of the dental adhesives (DA). These factors changed the results, as compared to the use of rigid models [34].

One of the factors that influences complete denture (CD) retention in the oral cavity is mucosal compression [27]. In our study, we used mandibular models to simulate the mucosa of the edentulous patient with greater retention problems [35]. Our simulation model followed the technique described by Johnson et al. [27], with modifications using new technologies. The materials used also followed the study conducted by Choi et al. [26].

The model in this study simulated oral mucosa, with the clinical application of resulting behaviors in the distribution of forces, as described by Choi et al. [26]. Artificial saliva was used due to its role in retention. According to Johnson et al., the saliva used in a DA retention study is of crucial importance as in vivo retention depends on it. The use of artificial saliva is important due to its viscosity, as compared to studies using water [36,37]. The hydration time of the models and DA was 10 min, using the method described by Flores-León et al. Saliva was maintained at 37 °C, reproducing the conditions of the oral cavity, as reported by Ikemura et al [30].

This study was conducted to compare the tensile strength of three original DAs and three white label DAs, in a simulation model using a CD with a corresponding DA. As in other DA retention tests, we used 200 mg of adhesive [34,38]. The tests were performed on a universal test machine, using a load of 10 N, to exert pressure and traction in the opposite direction at a cross-head speed of 5 mm/min. The maximum force was recorded, and the retention force was calculated, according to tests performed by several authors [27,30]. The measurements were performed after the DA application and the following 10 min of hydration [37]. This selected time interval was based on several articles described in the literature and, according to ISO 10873, for type 1, class 2 dental adhesives [31,37].

In 2013, Kore et al. evaluated the tensile strength of denture adhesives on denture base materials at time intervals of up to 24 h. Four denture adhesives were tested with 3 denture base materials: 2 heat polymerized (Lucitone 199 and SR Ivocap) and 1 visible light polymerized (colour stable Eclipse). Tensile strength was tested according to American Dental Association specifications 5 min, 3 h, 6 h, 12 h, and 24 h after adhesive application. All 4 adhesives exhibited higher bond strength than the control group. Fixodent, Super Poligrip and SeaBond had higher bond strength values than Effergrip. All adhesives had the highest bond strength after 5 min and the lowest after 24 h. The 3 denture bases produced significantly different results with each adhesive [34]. In accordance, we also evaluated the retention capacity of the DAs. The retention of the DAs was compared by brand, designation of strength of retention, and cost–benefit ratio in relation to the average gain of adhesion. The highest average retention force was obtained by KuKident Pro, which had a strong fixation designation, followed by Corega 3D Fixation, which had a strong designation (Table 6). In our study, KuKident Pro obtained a maximum retention value of 19.22 N. This DA had silica in its structure, which is a component that can change the viscosity of the paste and reduce humidity, leading to increased retention. KuKident Pro obtained the highest retention result, which was statistically significant in relation to all other brands, possibly due to the silica content. These results agree with those of Quiney et al.. In their study, the DA that obtained the best retention results was Fixodent^®^ due to the silica in its composition [35,36,37,38]. 

In 2017, Quiney et al. tested the effect of adhesives on the retention force of saddle-shaped partial dentures in the mandible. An in vitro model was developed, based on an anatomically accurate impression of a clinical case. Experimentally, the amount of adhesive was varied (0.2–1 g) and the traction force required for displacement was measured. Various commercial adhesives were then tested with optimal volume on the in vitro model. Using a 3D finite element model of the prosthesis, they examined how the forces required to displace the prosthesis varied as a function of the position of the force along the saddle length. They concluded that the use of adhesives significantly improved retention of the partial denture at the free end of the mandibular saddle, with the worst-performing adhesive increasing retention by a factor of nine, while the best-performing adhesive increased retention by a factor of twenty-three [38]. 

Corega^®^ Fixation 3D obtained a maximum retention force of 16.87 N and an average retention force of 13.05 N. These results differed from those of Mañes et al., in which the maximum retention force of Supercorega^®^ (equivalent to Corega^®^ Fixation 3D) was 19.6 N, and the mean retention force was 4.82 N. There was a significant difference between the mean values obtained in our study and the mean values achieved by Mañes et al. Flores-León et al. found that the average retention force of Corega^®^ Ultra Paste was between 12.233 N and 23.05 N, depending on the prosthetic bases used [35,37]. This result was in accordance with our study, in which the mean value was 13.05 N. KuKident^®^ Pro (extra-strong retention adhesive) and Corega^®^ Fixation 3D (strong retention adhesive) obtained the highest results.

Elgydium^®^ Fix was the most recent original DA brand and had no associated studies. It had the lowest average retention value, as compared to white label brands.

In this study, we compared the original brands with white brands as we had not found comparative studies between these DAs and, therefore, did not have reference studies, which is why we included these DAs and the cost/benefit ratios in our study. The retention disparity between KuKident^®^ Pro and Corega^®^ 3D Fixation has been well established in relation to generic DAs. The advantage of the generic brands used in this study was reduction in cost, in addition to being superior to Elgydium^®^ Fix, an original brand of DA.

## 5. Conclusions

Original brands of dental adhesives (Das) are adhesives with a greater retentive strength than generic DAs. However, Elgydium^®^ Fix (original brand) obtained the lowest values in this study.

Among the white label DAs tested, the Pingo Doce^®^ brand obtained the best results. The other white brand DAs were equivalent in terms of results. KuKident^®^ Pro was an extra-strong DA that had the highest retention of all the studied products. Corega^®^ 3D Fixation was the DA with the highest retention force in the strong category.

There was a difference between the original and white label brands in the DAs with extra-strong fixation. This difference was smaller among the DAs with strong fixation.

By purchasing a white label brand DA, it is possible to obtain a product at a much lower price, as compared to original DAs. Elgydium^®^ Fix was the DA with the highest cost and the lowest retention, even as compared to white label DAs.

A detailed cost–benefit analysis of DAs could be the subject of further study.

## Figures and Tables

**Table 1:** Denture adhesives used.

Adhesive Name	Types and Classes	Fixation Type	Composition	Company
Corega^®^ Fixação 3D	Type 1, Class 2-cream form	Strong	Calcium/Sodium PVM/MA Copolymer, Petrolatum, Cellulose Gum, Paraffinum Liquidum	GlaxoSmithKline^®^, Stafford Miller Ltd., Dungarvan Co. Waterford, Ireland)
KuKident^®^ Pro	Type 1, Class 2-cream form	Extra-strong	Calcium/Zinc PVM/MA Copolymer, Paraffinum Liquidum, Cellulose Gum, Petrolatum, Sílica	Procter & Gamble Technical Centres^®^, Ltd., Whitehal Lane, Germany
Elgydium^®^ Fix	Type 1, Class 2-cream form	Strong	Paraffinum Liquidum, Calcium/Sodium PVM/MA Copolymer, Cellulose Gum, Petrolatum, PVP, Isopropyl Palmitate, Isopropyl Myristate	Laboratórios URGO^®^ SL, Florida, Spain
Fixador de próteses (Continente^®^)	Type 1, Class 2-cream form	Strong	Paraffinum Liquidum, Cellulose Gum, Calcium/Sodium PVM/MA Copolymer, Petrolatum, Aqua, Polyethylene, Mentha Piperita Oil, Menthol, Limonene	Propack^®^,Gmbh, Ladenburg, Germany
Fixação Extra forte (Pingo Doce^®^)	Type 1, Class 2-cream form	Extra-strong	Cellulose Gum, Calcium/Sodium PVM/MA Copolymer, Paraffinum Liquidum, Petrolatum	Laboratórios Cosmodent^®^, Cantabria, Spain
Creme Fixador de próteses (Auchan^®^)	Type 1, Class 2-cream form	Extra-strong	Paraffinum Liquidum, Cellulose Gum, Calcium/Sodium PVM/MA Copolymer, Petrolatum, Silica, Aroma Menthyl Lactate	Ellipse^®^, Roubaix, France

**Table 2:** Adhesive retention compared by type of brand and brand.

Original Brand (n = 150)			White Label Brand (n = 150)			*t*-Test
11.16 (5.27)			5.92 (1.18)			t_(298)_ = 11.88 (*p* < 0.001)
Corega^®^ Fixação 3D (7.49–7.99) (n = 50)	KuKident^®^ Pro (7.29–8.96 ) (n = 50)	Elgydium^®^ Fix (7.90–8.46) (n = 50)	Continente^®^ (3.29) (n = 50)	Pingo Doce^®^ (3.29) (n = 50)	Auchan^®^ (3.30) n = 50)	ANOVA
13.05 (2.13)	16.16 (1.34)	4.27 (0.76)	5.24 (0.94)	6.71 (1.28)	5.80 (0.79)	F_(5.294)_ = 707.68 (*p* < 0.001)

Results presented as M(SD); price is in EUR.

**Table 3:** Comparisons of adhesive retention by fixation and brand.

Strong Fixation (n = 150)			Extra-Strong Fixation (n = 150)			*t*-Test
7.52 (4.19)			9.57 (4.84)			t_(298)_ = 3.90 (*p* < 0.001)
Corega^®^ Fixação 3D (7.49–7.99) (n = 50)	Elgydium^®^ Fix (7.90–8.46) (n = 50)	Continente^®^ (3.29) (n = 50)	KuKident^®^ Pro (7.29–8.96) (n = 50)	Pingo^®^ Doce (3.29) (n = 50)	Auchan^®^ (3.30) (n = 50)	ANOVA
13.05 (2.13)	4.27 (0.76)	5.24 (0.94)	16.16 (1.34)	6.71 (1.28)	5.80 (0.79)	F_(5.294)_ = 707.68 (*p* < 0.001)

Results presented as M(SD); price is in EUR.

**Table 4:** Comparisons of the adhesive retention by fixation and brand type.

Strong Fixation (n = 150)		Extra-Strong Fixation (n = 150)				
7.52 (4.19)		9.57 (4.84)		**ANOVA**		
Original Brand (n = 100)	White label Brand (n = 50)	Original Brand (n = 50)	White label Brand (n = 100)	Fixation	Brand type	Fixation x Brand type
8.66 (4.69)	5.24 (0.94)	16.16 (1.34)	6.26 (1.15)	F_(1.296)_ = 37.65 (*p* < 0.001)	F_(1.296)_ = 358.33 (*p* < 0.001)	F_(1.296)_ = 84.83 (*p* < 0.001)

Results presented as M(SD).

**Table 5:** Evaluation of mean retentive ability gains by mean price increase.

	Retention		
	β	IC 95%	*p*-Value
Price (in EUR)			
≈3.30 (White label brands)	5.92 (a)	5.70; 6.14 (a)	<0.001 (a)
Original Brands			
≈EUR7.74 (Corega^®^ Fixação 3D) (+4.44)	7.14	6.70; 7.57	<0.001
≈EUR8.13 (KuKident^®^ Pro) (+4.82)	10.24	9.80; 10.67	<0.001
≈EUR8.18 (Elgydium^®^ Fix) (+4.88)	−1.65	−2.09; −1.21	<0.001

R^2^ = 91.4%; (a) mean retention at baseline (intercept).

**Table 6:** The retention capacity of the adhesives in descending order, their characteristics, and their relation with price.

Adhesive Name	Type of Brand	Fixation Type	Price (in EUR)
KuKident^®^ Pro	Original Brand	Extra-strong	8.13€
Corega^®^ Fixação 3D	Original Brand	Strong	7.74€
Fixação Extra forte (Pingo Doce^®^)	White label Brand	Extra-strong	3.29€
Creme Fixador de próteses (Auchan^®^)	White label Brand	Extra-strong	3.30€
Fixador de próteses (Continente^®^)	White label Brand	Strong	3.29€
Elgydium^®^ Fix	Original Brand	Strong	8.18€

## Data Availability

The data that support the findings of this study are available from the corresponding author upon request.
